# Improving health through convergence science: reimagining our approach to solving the world's biggest challenges

**DOI:** 10.1093/pnasnexus/pgac007

**Published:** 2022-03-02

**Authors:** Victor J Dzau, Jenna L Ogilvie, Michael McGinnis

**Affiliations:** President, National Academy of Medicine, 500 Fifth Street, NW, Washington, DC 20001, USA; Publications Manager, National Academy of Medicine, 500 Fifth Street, NW, Washington, DC 20001, USA; Leonard D. Schaeffer Executive Officer, National Academy of Medicine, 500 Fifth Street, NW, Washington, DC 20001, USA

The three existential crises of our time—pandemic illnesses, including COVID-19; the impacts of climate change; and glaring and persistent health inequities rooted in centuries of structural racism—all devastating to human health—reflect a new reality that science and health must confront head on. The challenges society faces now, and the new crises that lie ahead, are increasingly complex and multifaceted, crossing disciplines, sectors, and geographies. Although the health impacts, individually and collectively, are stunning, responsibility for solving these challenges lies far beyond the health and medical arena. They are fundamentally connected to changes in our environment, our communities, our cultures, how we live and work, and society writ large.

**Figure 1:**
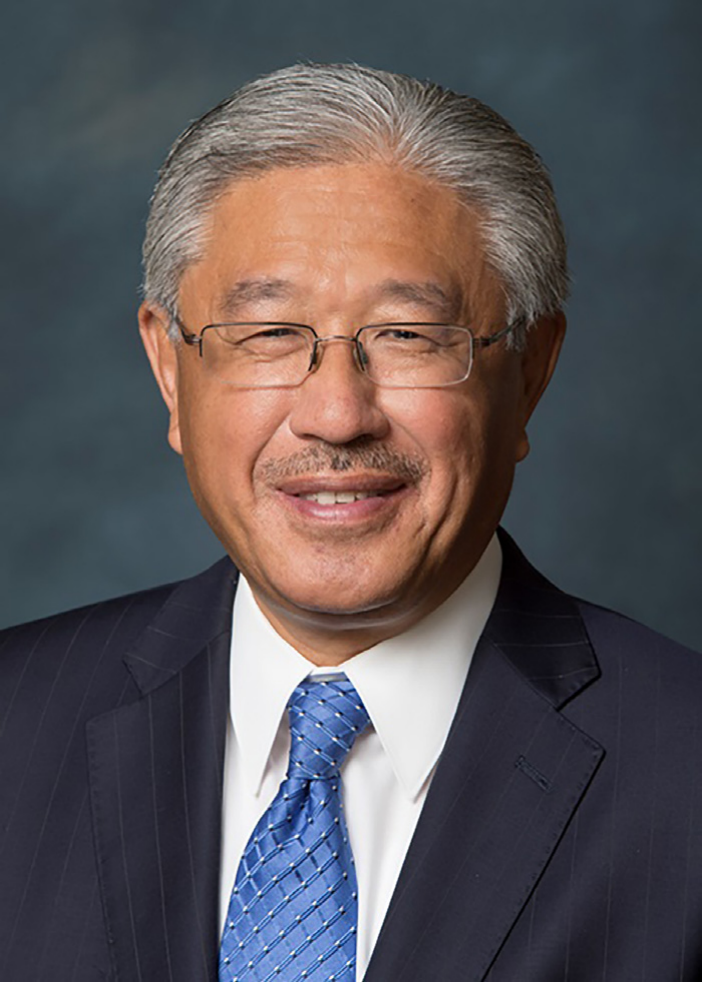
Victor J. Dzau

It is well-known, for example, that across society as a whole, health care accounts for only about 10–15% of health outcomes (1). Social and environmental factors such as access to nutritious food, safe housing, stable employment, and clean air contribute significantly to the overall health and well-being of individuals and communities. The future of health and health care is about the nexus of these factors across science and society. These convergences will shape both our greatest challenges and our greatest opportunities.

At the National Academy of Medicine (NAM), we are working to operationalize the best available scientific evidence to improve health and well-being for all. We also understand the need to adjust to the changing nature of the challenges before us, and to start thinking and working in different ways.

Convergence science is a critical pathway to understanding and engaging these complexities. A 2016 report from the Massachusetts Institute of Technology describes convergence science as “an approach to problem solving that integrates expertise from life sciences with physical, mathematical, and computational sciences, as well as engineering. . . (2)” This approach, “goes beyond collaboration . . . it is the integration of historically distinct disciplines and technologies into a unified whole that creates fundamentally new opportunities. . . (3)”

NAM embraces convergence science to transform health and medicine. However, it is essential to broaden its definition beyond the physical, mathematical, and engineering sciences. To improve population health outcomes, it will require the social, behavioral, economic, data, legal, political, and other sciences to generate research, policy, and implementation strategies. Indeed, the lead author has previously argued that population health must be approached as a convergence science to truly make progress, because “improving the health of populations requires an understanding of the myriad factors that influence health (4).” This includes looking beyond the biological notion of “good health” and understanding that health also encompasses elements like racial equity, social justice, and environmental sustainability. Leaders in the health and medicine arena must take an expansive view of their role and embrace their responsibility to advance societal good. Academic medicine itself should also examine how it operates and educates new clinicians, and consider how implementation of convergence science could spark transformative improvements, breakthroughs, and collaborations (5).

The NAM is practicing what we preach. For example, our Committee on Emerging Science, Technology, and Innovation examines cutting-edge innovations in health and medicine through the lens of their potential social, ethical, and legal implications. The committee has developed a preliminary multisectoral governance framework that will help guide future decision-making around the development and rollout of emerging technologies. The work of this committee will be continued in a consensus study, launching in 2022. Similarly, our recently launched Grand Challenge on Climate Change, Human Health, and Equity is a global effort that will harness bold thinking and radical, innovative, and collaborative partnerships to address climate change as both a public health and an equity crisis.

You could also say that convergence science is part of our DNA, as interdisciplinary research and practice is written in our bylaws. The NAM's Articles of Organization themselves stipulate that at least one-quarter of our membership be selected from fields outside the health professions.

COVID-19 has only further reinforced how interconnected we all are. When it comes to the crises we face, we truly are all in this together. We need solidarity, new partnerships, and new approaches in how we collaborate across borders, disciplines, and societies to dismantle the existential crises that face our nation and the globe.

These innovative approaches bring us to *PNAS Nexus*, a new journal dedicated to encouraging and sharing interdisciplinary scientific research. *PNAS Nexus* is the first of its kind, as it brings the sterling reputation of the *Proceedings of the National Academy of Sciences* together with an explicit focus on convergence science and a commitment to open access. *PNAS Nexus* is exactly the type of journal this moment in health and science needs—one that, by its charter, will bring together experts from across fields to creatively and collaboratively address entrenched and intractable problems, and then make these innovative solutions available to everyone. The democratization of science and health data is critical as we pursue interdisciplinary solutions and invite everyone to consider the health impacts of their decisions.

As we have seen during the COVID-19 pandemic and other global challenges, breaking down siloes is absolutely necessary to solve these existential threats. We are enthusiastic for the future of *PNAS Nexus*, as we know that the solutions to our greatest challenges are out there, waiting to be shared.
